# Environmental DNA and Hydroacoustic Surveys for Monitoring the Spread of the Invasive European Catfish (*Silurus glanis* Linnaeus, 1758) in the Guadalquivir River Basin, Spain

**DOI:** 10.3390/ani15020285

**Published:** 2025-01-20

**Authors:** Ruth Coya, Amadora Rodríguez-Ruiz, Álvaro Fueyo, Carlos Orduna, Laura Miralles, Ilaria de Meo, Trinidad Pérez, Juan Ramón Cid, Carlos Fernández-Delgado, Lourdes Encina, Yaisel J. Borrell, Carlos Granado-Lorencio

**Affiliations:** 1 Department of Functional Biology, Genetics, University of Oviedo, 33006 Oviedo, Spain; coyaruth@uniovi.es (R.C.); afueyo@taxusmedioambiente.com (Á.F.); miralleslaura@uniovi.es (L.M.); pereztrinidad@uniovi.es (T.P.); 2 Department of Plant Biology and Ecology, University of Sevilla, 41012 Sevilla, Spain; dora@us.es (A.R.-R.); granado@us.es (C.G.-L.); 3 Environment and Sustainability Area, Taxus Medio Ambiente, 33006 Oviedo, Spain; 4 EcoFishUS Research S.L.L., 41009 Sevilla, Spainilaria@ecofishus.com (I.d.M.); juanramon@ecofishus.com (J.R.C.); 5 Department of Zoology, University of Cordoba, 14071 Cordoba, Spain; ba1fedec@uco.es

**Keywords:** early detection, monitoring, echosounder, eDNA, Doñana National Park

## Abstract

The European catfish (*Silurus glanis* Linnaeus, 1758) was introduced into the Ebro Basin in Spain in 1974 for recreational fishing. Since then, the species has spread throughout the country’s river basins, reaching the Iznájar Reservoir (Guadalquivir River Basin) in 2011. This area is of great ecological and economic relevance, especially as it includes the Doñana National Park, one of the most important nature reserves in Europe. Recently, the presence of catfish has been reported in the lower reaches of the river. In this work, we used non-invasive vertical and horizontal hydroacoustic surveys and environmental DNA river sampling to unravel the actual distribution and dispersal pattern of the species in the lower Guadalquivir River. The hydroacoustic profiles and the species-specific detections by real-time PCR (qPCR) and droplet digital PCR (ddPCR) showed that these non-invasive methods allow the detection and quantification of catfish and provide valuable information on the species’ presence. We have confirmed the presence of catfish in most of the study area, including downstream areas of the Guadalquivir Basin. The results suggest the possibility of other isolated introductions and/or human-mediated movements of specimens, and imply that a coordinated catfish prevention and mitigation strategy is therefore urgently needed.

## Simple Summary

Introduced into the Ebro Basin in Spain in 1974, the invasive European catfish is now present in almost all Spanish river basins. This includes the Guadalquivir River, where it threatens native species and the ecological biodiversity of the Doñana National Park. This study provides valuable information on its current distribution using non-invasive methods such as hydroacoustics and environmental DNA and highlights the need for urgent control measures.

## 1. Introduction

Aquatic invasive species (AIS) are increasingly threatening ecosystems globally, causing biodiversity imbalances and having a substantial impact on economic enterprises [1]. Among the ecological impacts, AIS can significantly affect local biodiversity, potentially leading to the extinction of native species [2]. These impacts can arise from direct biotic interactions with the native fauna, such as competition and predation, as well as indirect changes in habitat conditions. In aquatic environments, organisms are characterised by strong trophic linkages that can be altered by introduced species. Invasive species in aquatic environments have strong and relatively constant ecological impacts [3].

**Figure 1:**
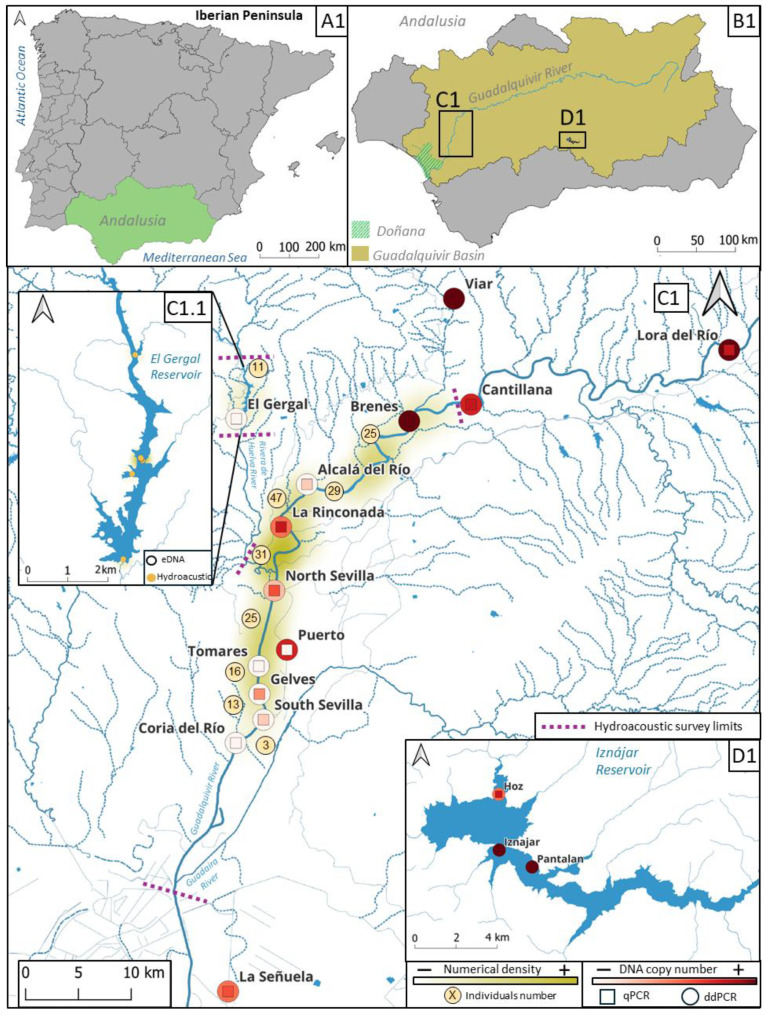
Detection and quantification of *Silurus glanis* using eDNA and hydroacoustic surveys in the Guadalquivir River Basin. (**A1**) Iberian Peninsula showing the Andalusian region. (**B1**) The Andalusian region, showing the Guadalquivir River Basin and including the Doñana National Park location. (**C1**) Detailed map of the Guadalquivir River Basin in the province of Sevilla showing the locations sampled for hydroacoustics and catfish eDNA (qPCR and ddPCR). (**C1.1**) Detail of the El Gergal Reservoir in Sevilla. (**D1**) Detailed map of the Iznájar Reservoir in the Guadalquivir River Basin, province of Córdoba. In this figure, the squares represent catfish eDNA qPCR quantification results while the circles around them represent catfish eDNA ddPCR quantification results. For both molecular methods, a heatmap from color white to color red dots gradient indicates the number of copies obtained. Hydroacoustic data (aggregate counts) is also represented by a heatmap from white to green indicating the numerical density of individuals greater than 1 m, while the number of individuals per stretch is given numerically in a yellow circle. In (**C1.1**) each individual is displayed as a yellow dot (some of them overlap in the middle) and white dots indicate the location of the two negative water samples taken for eDNA analyses.

Among invasive species in continental freshwater and low salinity (estuarine) bodies of water, the European catfish (*Silurus glanis*, Linnaeus, 1758) stands out as the largest fish in Europe excluding the diadromous sturgeon species [4]. Due to its piscivorous diet, the catfish poses a risk to native species and ecosystems [5,6,7,8]. Catfish were intentionally introduced in the Ebro Basin in Spain in 1974 and have since spread throughout the country’s river basins, primarily in the context of recreational fishing [6]. In July 2011, an individual of this species was captured in the Iznájar Reservoir, marking the first record of the European catfish introduced into the Guadalquivir River Basin (Figure 1) [9]. In 2015, the species was cited in the Rivera de Huelva River, a tributary on the right bank of the Guadalquivir River located more than 200 km downstream of the Iznájar Reservoir site [10]. Since then, the species has progressively appeared in different areas of the lower Guadalquivir River.

The lower area of the Guadalquivir River is home to a large number of aquatic species of great biological and economic value, making it the most important aquatic biodiversity hotspot in Andalusia, Spain [11,12,13,14,15]. The site covers 50,720 hectares of one of the largest wetlands in Europe and is the last area of largely undisturbed marshland within this ecosystem [16,17]. This region includes the lower Guadalquivir estuary, the southernmost river–estuary–delta system in continental Europe. Increasing human pressure and limited understanding of its dynamics have exacerbated socio-economic and environmental conflicts in recent years [18]. In addition, the biodiversity of the Doñana National Park and its associated ecosystems is threatened by intensive agriculture, which causes anthropogenic eutrophication of its waters [19], as well as by high groundwater use, pollution, hydrological infrastructure, climate change, and invasive species, which have significantly altered the ecological functioning of these systems [15]. As an invasive species, the catfish threatens biodiversity, as well as local economies and culture [20,21,22]. For this reason, it is important to study catfish presence, distribution, and dispersal patterns in ecologically sensitive areas such as the lower Guadalquivir. This has been the objective of the ’STOPSILURO’ (www.stopsiluro.es, accessed on 20 December 2024) project, launched in 2023 and targeting the development of control programs for this species that minimise its impact and prevent its spread in these areas of high ecological or socio-economic value.

Traditionally, aquatic species monitoring has depended on visual detection and often the capture of organisms. However, these methods are costly and labour-intensive [23]. In recent years, there have been significant advances in technologies for remote and early detection of invasive species. Hydroacoustics has proven to be a highly suitable tool for the quantitative characterisation of fish populations and their spatio-temporal distribution, as it allows large volumes of water to be sampled with great efficiency and high speed of data acquisition, which is why its use has increased in recent decades [8,24,25]. Regarding early detection, environmental DNA (eDNA) is considered one of the most promising analysis and management tools due to its high sensitivity in species detection [26,27,28].

The potential of hydroacoustic methodology as a useful tool for fish species management and control programmes, including exotic species, has been shown [8], although hydroacoustic equipment currently lacks the ability to identify species other than by body size or habitat distribution. However, in ecosystems where there is a clear differentiation of species based on their size, or where fish communities are monospecific or there is clear habitat segregation according to species, modern hydroacoustic techniques have been used as useful evaluation tools for fish species [24,25,29]. Regarding the European catfish, the possibility of using hydroacoustic techniques to evaluate and monitor its presence and abundance is of great relevance given the difficulty of catching this species with traditional fishing methods, which often leads to its underestimation [30]. Catfish are characterised by a high growth rate, especially during the first year of life [31,32]. Thus, the large size reached by this species at an early age, combined with the smaller size of the other species present in the study area, enables adult catfish specimens to be differentiated from the rest of the species using hydroacoustic methods.

Previous studies have successfully applied the environmental DNA (eDNA) methodology for the specific detection of catfish, demonstrating its sensitivity [33,34]. Parrondo et al. [35] proposed the combination of comprehensive reviews of citizen alerts with verifications based on specific molecular techniques as a rapid, cost-effective, and truly accessible strategy for the early detection of catfish in Spain. This could serve as a preliminary step before more comprehensive information phases in management plans addressing invasive species [35]. Currently, it is feasible to design species-specific primers for rapid detection, even when DNA is partially degraded. DNA degradation can pose a challenge in natural environments or environmental samples, but advances in primer design techniques and DNA amplification overcome these difficulties [36]. By reducing the time and resources needed for analyses, the implementation of conservation and natural resource management strategies is facilitated, along with the study of biological populations and communities [36,37].

Environmental DNA allows not only detection but also quantification of the species studied through two molecular techniques: the quantitative polymerase chain reaction (qPCR) and the droplet digital PCR (ddPCR) [38]. The qPCR measures the copy number of the amplified target fragment using a calibration curve derived from serial dilutions of a sample with a known concentration. The threshold fluorescence level is computed from initial cycles, and the cycle number (CT value) correlates with the sample’s template copy number [39]. Therefore, quantification via qPCR relies on an indirect measure from a calibration curve, limiting its precision and reproducibility. In recent years, a new quantitative method, ddPCR, has emerged, directly quantifying DNA without reference curves. This involves partitioning the sample into thousands of droplets and independently conducting a PCR in each, detecting fluorescence at the process’s conclusion, as an end-point measurement. Previous studies have demonstrated the effectiveness of both qPCR and ddPCR techniques in quantifying DNA concentrations of target species in eDNA from mesocosm experiments with known abundances and biomasses [40,41,42]. Estimates via ddPCR show less variation than qPCR, suggesting they can calculate eDNA concentration more accurately, enhancing the ability to estimate species abundance/biomass in eDNA studies [42,43]. However, not enough studies have been conducted on environmental samples to determine species distributions in natural habitats and the potential problems and benefits of both techniques.

Environmental DNA-based technologies, coupled with the use of echosounders, can provide accurate and early detection of invasive species such as catfish, thus contributing to comprehensive monitoring of biodiversity and environmental impacts in different aquatic environments [44]. In this study, a dual mapping strategy was implemented along the Guadalquivir Basin, using an environmental DNA detection method for monitoring catfish based on the molecular techniques of real-time quantitative PCR (qPCR) and droplet digital PCR (ddPCR). The objective of this study was to determine the range of expansion of European catfish in the lower Guadalquivir and also to assess the complementarity and potential synergies between eDNA analysis and hydroacoustic methods for monitoring aquatic invasive species in riverine environments.

## 2. Materials and Methods

### 2.1. Hydroacoustic Study

#### 2.1.1. Hydroacoustic Survey

The acoustically surveyed area included three main zones, identified based on river morphology. The Alcalá del Río dam represents the final barrier before the Guadalquivir River flows into the sea. Therefore, we considered the section of the river upstream of the Alcalá del Río dam, extending to the Cantillana dam, as a separate area. This section forms the Alcalá del Río Reservoir (Figure S1). Next, we surveyed the section of the Guadalquivir River downstream of the Alcalá del Río dam up to its confluence with the Guadaíra River, covering a total of 45 km. The northern portion of this section, from the Alcalá del Río dam to South Sevilla, is narrower, with a width of approximately 150 m, while the southern portion, from South Sevilla to the confluence with the Guadaíra River, is wider, averaging around 300 m. Finally, we included the El Gergal Reservoir, which connects to the Guadalquivir River through the Rivera de Huelva tributary near North Sevilla.

Sampling was conducted using an outboard boat, with navigation always carried out upstream and sailing speed maintained at a steady 4 knots. The sampling was conducted throughout the month of June of 2023, during daylight hours, between 8 a.m. and 9 p.m. While the European catfish is primarily nocturnal, the lack of administrative permissions for night-time navigation, due to the unsafe conditions in the surveyed river area, made sampling during night impractical. Nonetheless, studies have shown that it can be effectively detected during the day because the higher daytime temperatures keep them active [8,45,46]. For the hydroacoustic surveys, we used a Simrad EK60 echosounder (Simrad Kongsberg Maritime AS, Horten, Norway) with a split-beam circular transducer operating at 200 kHz (ES200-7 C). The transducer was installed on a stainless-steel frame (utility model number ES1279955, EcoFishUS and University of Sevilla, Sevilla, Spain). The structure was mounted on the side of the boat, providing the equipment with good stability during sailing.

The hydroacoustic survey was conducted both vertically and horizontally to cover the entire ecosystem, including the surface and deepest zones. The surface water layer was surveyed using horizontal hydroacoustics, with the transducer positioned 120 cm below and parallel to the water surface. This ensured that the acoustic beam’s opening angle insonified a representative volume of the first 2 m of depth, avoiding the potential effects of engine noise and the near-field effect [47,48,49]. Zones with a depth greater than 2 m were surveyed using vertical hydroacoustics, with the transducer positioned 40 cm below the water surface and oriented towards the bottom. This configuration ensured that measurements taken below 2 m were reliable and unaffected by engine noise or the near-field effect [47,48,49]. The survey was conducted while navigating upstream in zigzag trajectories, except in areas where the river was too narrow for horizontal hydroacoustic surveys to insonify a representative volume. In such cases, parallel transects along the shores were performed, ensuring the insonification of the entire area.

Before sampling, the acoustic unit was calibrated using a calibration copper sphere following the standard calibration method [50]. The pulse duration was 0.256 ms, and the pulse mode was set to the maximum offered by the echosounder for the depths studied. The position of the transducer was geo-referenced in real time using an Emlid Reach RS2 GPS (Emlid Tech Kft., Budapest, Hungary) placed on the transducer structure.

#### 2.1.2. Hydroacoustic Data Post-Processing

The data stored during the echo surveys were processed using the hydroacoustic analysis software Sonar5 Pro 608.43 [51]. Prior to processing, the bottom line was set in all sampling files to be able to discriminate between fish and bottom echoes. Subsequently, various sources of noise that could lead to errors in fish detection, such as bubbles, vegetation, patches of phytoplankton, zooplankton aggregations, and other noise sources were removed.

#### 2.1.3. Catfish Detection

The acoustic signals were analysed to detect specimens that could potentially be catfish based on their size. To differentiate catfish from other fish species in the study area, a threshold of 1 m in total length was established for detected individuals. Among the species cohabiting the study area, some can reach lengths exceeding 50 cm, including the carp (*Cyprinus carpio*, Linnaeus, 1758), southern Iberian barbel (*Luciobarbus sclateri*, Günther, 1868), striped mullet (*Mugil cephalus*, Linnaeus, 1758), thinlip grey mullet (*Chelon ramada*, Risso 1826), twaite oliveshad (*Alosa fallax*, Lacépède, 1803), largemouth bass (*Micropterus salmoides*, Lacepède, 180), or European eel (*Anguilla anguilla*, Linnaeus, 1758) (Table S1) [6,52,53,54]. While this size threshold excludes smaller catfish, it minimises potential overlap with other species, ensuring that all detected specimens are unequivocally catfish, which is the primary focus of the survey.

For the analysis of transects from vertical surveys, the echo counting method based on single echo detection (SED) was used. The Target Strength (TS)-length relationship equation published by Love [55] and included in the standard UNE-EN 15910 [56] was employed for the TS-size conversion. In this case, the equation relates the TS to the total length (TL) of the fish. Size ranges with TS greater than −25.81 dB correspond to sizes greater than 1 m, so they were counted as catfish. All detections corresponding to individuals within these size ranges were georeferenced using the information stored during simultaneous GPS-acoustic surveying.

For the analysis of transects from horizontal surveys, the trace counting method based on fish tracks was employed. The equation used for the TS-size relationship was developed by Kubečka and Duncan [57], as specified in the UNE-EN 15910 standard. This equation considers the different orientations in which a specimen can be insonified during horizontal surveys. Unlike vertical surveys, where the fish is almost always insonified dorsally, in a horizontal survey, a fish can be insonified in different positions, ranging from head-to-tail to lateral. The head-to-tail position generates a lower TS by exposing less of the surface area of the fish to the acoustic beam than the lateral position, where a larger surface area is exposed. However, both positions correspond to the same size, so it is crucial to consider the orientation of the fish relative to the acoustic beam. In the track analysis, the program assigns the specific size corresponding to the specimen that has generated a particular trajectory based on the angle of the trajectory relative to the acoustic beam. Each track with a size equal to or greater than 1 m was manually checked using the analysis software tools, which provide a visual representation of the tracks across the different spatial axes to verify they originate from a catfish. As in vertical sampling, all catfish detections were georeferenced in the study area.

### 2.2. The eDNA Procedures and Analyses

#### 2.2.1. Field Sample eDNA Collections

Water sampling was conducted in rivers and reservoirs of the Guadalquivir Basin located in the provinces of Sevilla and Córdoba in southern Spain (Figure S2) between 25 and 29 June 2023. The sampling sites were mostly located in areas that are exposed to sunlight for many hours and have no vegetal canopy to protect them from direct UV radiation, which can accelerate DNA degradation. Sampling was conducted during daylight hours but was structured to avoid the central parts of the day (12:00 to 15:00), as these coincide with the peak solar radiation and higher temperatures. A total of 34 samples of 2 litres each were collected from 17 sampling sites (Figure S2). Sampling points were clustered into 3 study areas: positive control area (Iznájar, Pantalán, and De la Hoz), upper river area (Viar, El Gergal, Lora del Río, Cantillana, Brenes, Alcalá del Río, and La Rinconada) and lower river area (North Sevilla, Puerto, Tomares, South Sevilla, Gelves, Coria del Río and La Señuela). Niskin-type oceanographic bottles were used to collect water samples to a depth of at least one metre to avoid the shallowest layer (without touching the bottom), which is the hottest and most affected by solar radiation. A HannaR HI98494 (Hanna Instruments, Woonsocket, RI, USA) worked to measure these parameters in the water column at 1–2 m avoiding contact with the sediment, including pH, oxidation-reduction potential (mV), dissolved oxygen percentage (%), dissolved oxygen concentration (mg/L), electrical conductivity (µS/cm), total dissolved solids (ppm), turbidity (NTU), and temperature (°C), during sampling (Table S2).

Sampling was performed using sterile equipment and disposable nitrile gloves. All the materials were sterilised between sampling points using 10% *v*/*v* commercial bleach (final [Cl^−^] 0.3% *v*/*v*). Any residual bleach was then rinsed off with distilled water and the Niskin bottle was then rinsed again with reservoir water at the next sampling point. Benzalkonium chloride (BAC) was added to the samples to preserve the DNA using a pipette with sterile and filtered tips to a final concentration of 0.01% *w*/*v* BAC in the sample [58,59]. Two samples were collected at each sampling site (R1 and R2) and sampling was carried out attempting to separate the localities by a minimum of 2 km whenever possible. After sampling, the water samples were stored in sterile 2 L graduated bottles (REF. 407008.O) (Deltalab, Barcelona, Spain) at 4 °C until further processing.

#### 2.2.2. Filtration and DNA Extraction

Water samples were filtered within 24 h of collection in a decontaminated laboratory using a filtration ramp with three filtration units (Labbox Labware SL, Barcelona, Spain). Two litres per sample were filtered. Sample (2 L in total): Sampling replicate 1 (1 L) − Subsamples R1_1 (0.5 L) + R1_2 (0.5 L)/Sampling replicate 2 (1 L) − Subsamples R2_1 (0.5 L) + R2_2 (0.5 L). We filtered 2 L of each point, with 1 L per sample (0.5 × 4), using a dual-filter strategy to avoid clogging. The filters used were a cellulose nitrate membrane filter (CN) with a 1.2 μm pore size (SARTORIUS Cellulose Nitrate Filter 11403—50----ACN) and, below, a second CN filter with a 0.20 μm pore size (PRAT DUMAS France MCNE-247-100). Filtration equipment was sterilised with 10% *v*/*v* commercial bleach solution for 10 min and rinsed twice with sterile distilled water between samples. Filters were folded using sterile tweezers and placed in bead tubes provided by the PowerWater^®^ Kit (Qiagen, Hilden, Germany) and stored at −20 °C until DNA extraction. Filtration negative controls were run once a day between the samples to check for contamination (n = 6). Environmental DNA was extracted using DNeasy PowerWater^®^ Kit (Qiagen, Hilden, Germany) following the manufacturer’s instructions in a dedicated clean room for the processing of eDNA samples. Three extraction negative controls were added.

#### 2.2.3. The qPCR and ddPCR Analyses

Primer3 software implemented in Geneious Prime^®^ 2023.1.1 [60] was used for primers and probe design (see Supplementary Materials for more details). Two specific primers and a probe (FAM-labelled) were developed for a fragment of the 12S rRNA mitochondrial gene of the genus *Silurus*: Sil1-F (5′-TTTTCCCCGCCTATATACCGCC-3′) Sil1-R (5′-CTTCGGGCACTTACTTTCAAGG-3′) and Sil1-probe (**FAM-AACGTCAGGTCGAGGTGTAGCGTACG-MGB).

After a strict validation of the primers and probe (SI.1), the qPCR reactions were conducted in a dedicated pre-PCR laboratory where bench surfaces were sterilised with UV radiation. Each PCR contained 6 μL of template DNA, 1.2 μL of each primer (10 μM), 0.8 μL of probe (5 μM), 10 μL of TaqMan^®^ Environmental Master Mix 2.0 (Thermo Fisher Scientific, Waltham, MA, USA), and DNase/RNase-free water to a final volume of 20 μL. Each PCR plate contained a standard curve consisting of a 1:10 dilution series to generate a standard curve of known amounts of DNA. Three technical (PCR) replicates were performed for each biological sample, resulting in a total of twelve replicates per sampling point. In addition, 3 no template controls (NTCs) per plate were included, as well as filtration and extraction controls. The filtration, extraction and technical controls returned negative results. Samples were run on an Applied Biosystems 7900HT Fast Real-Time PCR System (Thermo Fisher Scientific) under the following thermal cycling conditions: 2 min at 50 °C, 10 min at 95 °C, 50 cycles of 15 s at 95 °C, and 60 s at 60 °C. Sequence Detection Software SDS 2.4 (Thermo Fisher Scientific) was used to analyse the results. To include locations/sites for the rest of the analyses, two of the three technical replicates, besides one of the two subsamples (e.g., R1_1 (0.5 L) + R1_2 (0.5 L)) in at least one of the sites replicates (R1 or R2), should reveal catfish eDNA (as advised following a precautionary framework recommended by Goldberg et al. [61]).

Further optimisation and validation of the ddPCR assay were performed (SI.1). In this case, and due to logistical restrictions, subsamples (e.g., R1_1 (0.5 L) + R1_2 (0.5 L)) were pooled, and for each location/site, two replicates (Replicate 1 and Replicate 2) were analysed. Each ddPCR reaction mix (20 μL) comprised 10 μL of sample, 900 nM of each primer, and 250 nM of TaqMan probe, completed with Supermix for Probes (No dUTP) (1863023) (Bio-Rad, Hercules, CA, USA) at a concentration of 1×. This mixture was then combined with Bio-Rad’s droplet generation oil and divided into 15,000–20,000 droplets using the QX-100 droplet generator (Bio-Rad). Individual sample droplets were placed separately into each well of a 96-well PCR reaction plate. PCR was performed in the sealed 96-well plate using the Bio-Rad T-100 thermocycler (Bio-Rad). The final PCR conditions were 10 min at 95 °C, 40 cycles of denaturation for 30 s at 94 °C, and extension for 90 s at 57 °C with a temperature ramp of 2 °C/second, followed by 10 min at 98 °C, and a hold at 4 °C until plate reading. After PCR amplification, the plate was transferred to the Bio-Rad QX-200 droplet reader (Bio-Rad). Bio-Rad’s QuantaSoft software, version 1.7.4.0917, was used to quantify copies of the target DNA. To include locations/sites for the rest of the analyses, at least one of the site replicates (R1 or R2) should reveal catfish eDNA.

### 2.3. Mapping and Statistical Analyses

For the map graphics, we used QGIS v. 3.32.3 Lima (https://qgis.org/en/site/, accessed on 20 December 2024) with hydrography data from the Spatial Reference Data of Andalusia, Institute of Statistics and Cartography of the Government of Andalusia. Water physicochemical parameters were assessed for collinearity using a correlation matrix in R software v. 4.3.3. [62]. As a result, the following variables were dropped from the final regression analysis: pH, dissolved oxygen percentage (%), electrical conductivity (µS/cm), and total dissolved solids (ppm). Pearson correlation analyses [63] were conducted between log-transformed total qPCR and ddPCR eDNA copy numbers by site.

Principal component analysis (PCA) [64,65] was performed on physicochemical variables and qPCR and ddPCR catfish eDNA values using PAST V.4.17 [66]. A PCA was also performed using the total catfish eDNA values obtained by qPCR and ddPCR per site, the hydroacoustic catfish detections (aggregate counts) in river sections between 1 and 10 km upstream of the eDNA sampling sites (the mid-point between replicates R1 and R2 was taken as the reference point), and finally the distances from each eDNA sampling site to the river mouth (km). Stepwise linear regression analyses were also performed to identify associations between log-transformed response variables (eDNA ddPCR and qPCR copy numbers), and potentially explanatory variables in river and reservoir habitats wherever possible. Moreover, and despite temporal and spatial heterogeneity between the two detection methodologies used in this work, Fisher’s exact test (presence/absence) [67] and Spearman’s rank correlation were used to assess possible relationships between the abundance of catfish detected by hydroacoustics (estimated as counts upstream of eDNA sampling points as in the previous analyses), molecular techniques (qPCR and ddPCR catfish eDNA values), and distances from the river mouth (km).

## 3. Results

### 3.1. Hydroacoustic Survey Results and Spatial Distribution of S.glanis

Both vertical and horizontal surveys confirmed the presence of catfish in the lower Guadalquivir as well as in the El Gergal and Alcalá del Río Reservoirs (Figure 1). A total of 200 individuals were positively identified as adult catfish measuring 1 m or more. In the El Gergal Reservoir, a total of 11 individuals were detected, with the highest number in the dam area (9 individuals); in the Alcalá del Río Reservoir, 54 catfish detections were evenly distributed across the study area and, overall, were greater than in the El Gergal Reservoir. The highest number of catfish was detected in the river, particularly in the stretch between the Alcalá del Río dam and North Sevilla, with 78 detections decreasing progressively downstream, 54 detections in the rest of the Sevilla area, and 3 in the Coria del Río area, the last location where catfish was detected. Most of the detected individuals fell within the size range of 1.0 to 1.2 m (Figure S3).

### 3.2. Environmental DNA-Specific Detections, Quantifications and Spatial Distribution of S.glanis

The mean and standard deviation values for each physicochemical parameters from the sampling locations were estimated (Table S2): pH (µ = 8.2 ± 0.45), oxidation-reduction potential (ORP) (µ = 33.2 ± 9.55 mV), dissolved oxygen percentage (µ = 71.8 ± 42.80%), dissolved oxygen concentration (µ = 5.5 ± 3.23 mg/L), electrical conductivity (µ = 1769.2 ± 1118.84 µS/cm), total dissolved solids (µ = 885.2 ± 561.06 ppm), turbidity (µ = 0.9 ± 0.59 NTU), and temperature (µ = 28.6 ± 1.92 °C) showing high heterogeneity among sampling sites for some of the variables and evident correlation between some of them (Figure S4).

In the PCA analysis, PC1 and PC2 accounted for 74.8% of the total variability based on the eigenvalues obtained (Figure S4). Stepwise linear regression analyses with logarithmic transformation were performed among the variables, showing that in rivers, ddPCR copies were indeed positively associated with dissolved oxygen (mg/L) (*p*-value < 0.01) and negatively associated with turbidity (NTU) (*p*-value < 0.01). In reservoirs, copies detected by qPCR were also weakly negatively associated with turbidity (NTU) (*p*-value < 0.05) (Table S3).

Overall, both molecular assays (qPCR and ddPCR) demonstrated specific detection of catfish. There was no detection in any of the negative technical controls using either methodology. All filter and extraction controls showed no amplification, indicating the absence of contamination at all stages of the study. The qPCR data reported positive catfish detection in 13 sites (76.5%), while we obtained 11 positive sites with ddPCR (64.7%) from the 17 sample sites (Figure S5). A comparison of the results shows that 76.5% of the sites were concordant in terms of the presence of catfish eDNA between the two techniques tested. The qPCR results showed an average of 5.5 × 10^5^ catfish eDNA copies (ranging from 108.2 to 3.4 × 10^6^). Using the ddPCR methodology, the values for accepted generated droplets were on average 17,375 and ranged from 12,284 to 19,592. From this, an average of 454.5 positive catfish eDNA ddPCR copies were estimated (range: 60 to 1260 copies). Statistical analyses revealed a significant positive correlation between the logarithmically transformed copy number data obtained by qPCR and ddPCR for catfish environmental DNA (*p*-value < 0.01) (Figure S6).

The spatial distribution of catfish eDNA concentrations is shown in Figure 1. The *S. glanis* eDNA quantification results were higher at the sampling sites of the “positive” area within the Iznájar Reservoir (Iznájar, Pantalán, and Hoz), where all sites were positive for the presence of catfish (100%) with the highest quantification data of the study for qPCR (average 1.7 × 10^6^ copies) and ddPCR assays (average 773 copies) (Figure 1 and Figure S5). The upper river area, above the Alcalá del Río dam (Viar, Lora del Río, Cantillana, Brenes, El Gergal, and Alcalá del Río), had five out of six catfish eDNA qPCR positive sites (83%) (negative detection was found for El Gergal) with an average of 3.3 × 10^5^ copies detected. However, one of them, Alcalá del Río, was positive for only one qPCR replicate. For ddPCR, four out of six sites were positive (67%), with an average of 477 copies detected, while negative results were found for El Gergal and Alcalá del Río (Figure 1).

In the lower river study area downstream of the Alcalá del Río dam (La Rinconada, North Sevilla, Puerto, Tomares, Gelves, South Sevilla, Coria del Río, and La Señuela), there were qPCR detections for catfish eDNA (average of 6.9 × 10^4^ copies detected) in five out of eight sites (62%) (La Rinconada, North Sevilla, South Sevilla, Gelves, and La Señuela) (Figure 1 and Figure S5). However, for Gelves and South Sevilla, catfish eDNA was detected in only one qPCR replicate. Using the ddPCR method, we obtained four out of eight catfish eDNA positive sites (50%) (La Rinconada, North Sevilla (one positive ddPCR replicate), Puerto and a location further south, La Señuela) with an average of 192 eDNA copies (Figure 1 and Figure S5).

**Figure 2:**
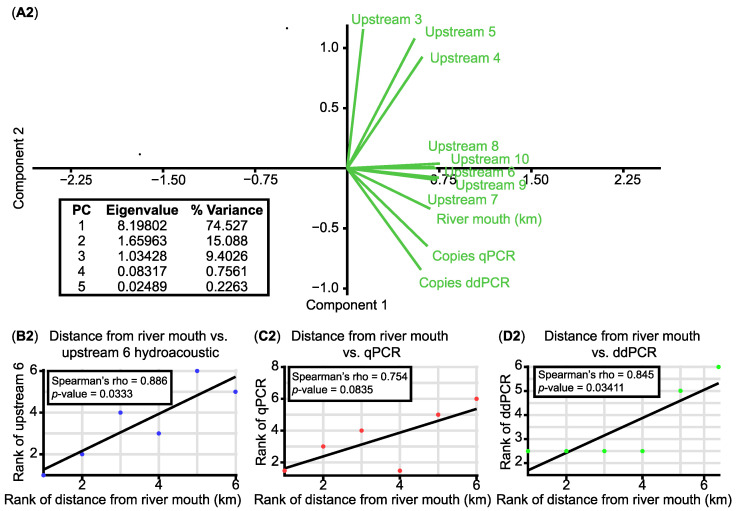
The abundance patterns of *Silurus glanis* using hydroacoustics and catfish eDNA qPCR/ddPCR dual mapping. (**A2**) Principal component analysis (PCA) biplot based on the correlation matrix of catfish eDNA qPCR and ddPCR copies in 2 L of water, hydroacoustic catfish detections (aggregated counts) upstream of the eDNA water sampling points ranging from 3 to 10 km and finally distances from river mouth (km) for each of the values. Spearman rank correlation test plot between (**B2**) distance from the river mouth and the hydroacoustic detection at 6 km of eDNA sampling, (**C2**) distance from the river mouth and the eDNA qPCR detections, and (**D2**) distance from the river mouth and the eDNA ddPCR detections. Different colored dots represent different correlation tests.

Principal component analysis enabled exploratory integration of catfish abundance data from hydroacoustics vs. eDNA qPCR/ddPCR data (Figure 2). For analysis, hydroacoustic catfish detections (aggregated counts) were estimated in river sections in a range from 1 to 10 km upstream of the eDNA sampling points. The resulting PCA biplot showed a positive correspondence (narrow angles, same quadrants) between catfish eDNA qPCR/ddPCR copies, distances to the river mouth, and some hydroacoustic aggregated counts at distances greater than 6 km upstream of each eDNA sampling point (Figure 2). The PC1 and PC2 eigenvector values accounted for 74.5% and 15.1% of the data variance, respectively (a total of 90% of the total variance).

Positive and significant correlations were found between distances from the river mouth and catfish abundance detected by the different techniques, implying higher catfish abundance in upper areas within the Guadalquivir Basin (Figure 2). Spearman’s rank correlation analyses showed a significant positive correlation between the abundance of catfish detected by hydroacoustics at 6 km and 7 km upstream sections of the eDNA sam-pling points and distances to the river mouth (rho = 0.89, *p*-value = 0.033) (Figure 2B2). There was also a significant positive correlation between catfish abundance detected by ddPCR and distance from the river mouth (rho = 0.85, *p*-value = 0.034) and positive and marginally, but not significant, relation with the qPCR data (rho = 0.75, *p*-value = 0.083) (Figure 2C2,D2). A simple Fisher’s exact test (presence/absence) did not reveal significant relationships between the hydroacoustics and the two molecular technique detections (*p*-value > 0.05). Spearman’s rank correlation tests indicate positive but not statistically significant correlations (*p*-value > 0.05) between the hydroacoustic catfish detections and the qPCR/ddPCR copies.

## 4. Discussion

It has been claimed that the presence of *S. glanis* in the Guadalquivir River Basin may consist of two populations: one confined within the limits of the Iznájar Reservoir, located in the Genil River Basin, a tributary on the left-hand side of the main river, and a second population centred in the Guadalquivir River [10,68]. These populations are separated by more than 200 km of river, with no presence of the species along this distance. This could suggest that the Guadalquivir River has possibly experienced two separate human introductions of the species within the last ten years. Although the presence of the species is always concerning, its appearance in the lower area of the river is more alarming due to the ecological and economic importance of the invaded area.

### 4.1. Hydroacoustic Surveys Are a Useful Detection Tool for Uncovering Adult S.glanis Presence

The results obtained in this work using hydroacoustic surveys demonstrate the effectiveness of this method as an early detection tool for catfish in aquatic ecosystems. This is particularly relevant because this species is difficult to capture using standardised techniques in ecosystems like the one studied [30]. Consequently, its presence is usually reported years after its introduction, when the population has already reached high densities. This delay complicates the implementation of effective management plans or eradication efforts [8,69].

The large size reached early by catfish [7,31,32,70,71,72], combined with the size structure of the other species present in the surveyed area, allows for the differentiation of catfish specimens greater than 1 m TL from the rest of the species. This selection ensures that smaller catfish present in the area were not accounted for, which consequently results in the abundance of catfish in the surveyed area being higher than the abundance represented by the georeferenced specimens in the study. Nonetheless, this approach was crucial for accurately defining the species’ current expansion area and achieving the study objectives.

Hydroacoustic methodology has demonstrated significant potential as a tool for managing fish farms and monitoring exotic species [29,73,74]. As highlighted by Encina et al. [8], traditional netting methods often underestimate catfish populations due to the difficulty of capturing this species, with the hydroacoustic methodology proving to be a relevant tool for evaluating the species’ presence, especially in the early stages of an invasion.

### 4.2. Invasive S. glanis Can Be Detected Using Environmental DNA

Environmental DNA is a cost-effective and highly sensitive method that is widely used to improve our understanding of species distributions [60,75,76,77]. Despite this, it has been claimed that a more complete understanding of the “ecology” of eDNA (the origin, state, transport, and fate of extra-organismal genetic material) is essential to maximise its effective application in conservation and research [78]. There is currently no model that can accurately predict the location or abundance of fish from eDNA concentrations alone [79]. The eDNA sampling conducted in the Iznájar Reservoir was used here as a positive control for catfish detection in the field. This reservoir was, presumably, the entry point of *S. glanis* into the Guadalquivir area in 2011, and a high abundance of the species in the reservoir has been reported—officially and unofficially—since then [9,10,68]. All sampling points within the reservoir were positive when using the qPCR and ddPCR methods and two of them, the central ones (Figure 1), had the highest relative DNA copy number abundances in the study, confirming the effectiveness of eDNA for detecting *S. glanis* and suggesting a relationship with catfish abundance.

The section of the river upstream of the Alcalá del Río dam, including Alcalá del Río, Brenes, Cantillana, Lora del Río, Viar, and El Gergal, revealed the presence of catfish eDNA copies, with the exception of the El Gergal Reservoir (Figure 1). In this last locality, no positive results were obtained with any of the molecular techniques used, even though catfish are known to be present in the area [80], as confirmed by the hydroacoustic survey with a low number of individuals (Figure 1). Due to logistical constraints caused by a severe water shortage (<20% of capacity), only two close points (270 m apart) on the south-west bank of the reservoir were sampled for eDNA (Figure 1C1.1). The largest number of specimens detected by hydroacoustic were located in the northern area (Figure 1C1.1). As a result, there is a high probability that a site containing traces of catfish genetic material has not been sampled.

The river section (downstream of Alcalá del Río dam) including La Rinconada, North Sevilla, Puerto, Tomares, South Sevilla, Gelves, Coria del Río, and La Señuela was where the ddPCR and qPCR detection patterns showed more divergence in detectability. The ddPCR detected catfish eDNA only in the northern river locations (La Rinconada, North Sevilla, Puerto), whereas the qPCR also detected eDNA copies in South Sevilla and Gelves but not in the Port of Sevilla (both techniques detected catfish eDNA in La Señuela). As previously explained, South Sevilla and Gelves were the only two localities in this river section where replicates R1 and R2 yielded different results (only one of the replicates was positive in both cases). Nevertheless, it is relevant to report, regardless of sampling replication, since eDNA presence cannot be ignored when technical artefacts can be discarded. However, these qPCR results should be interpreted with caution, as recommended by Goldberg et al. [61]. When initial DNA concentrations are very low, discrepancies between qPCR replicates and potential false negatives are expected [81,82,83]. The port area, as a hub of activity, is probably susceptible to higher levels of environmental contamination, including PCR inhibitors, which could explain the failure of qPCR (in both replicates) while ddPCR successfully detected catfish.

Catfish eDNA was also detected at La Señuela, 30 km downstream of Coria del Río, the last site where the species was detected. This sampling site is located near the main riverbed, where the channels are supplied with water from a large pond (Balsa de Melendo; 36°57′ N; 6°02′ W) situated 12 km east of the Guadalquivir River’s main riverbed. A decomposed specimen of catfish was found by the authors in the vicinity of the Melendo pond in December 2023, perhaps providing additional evidence of the progress of the colonisation/invasion process. Transport of genetic material from the last river sites where the species was detected seems highly unlikely. Future studies should also focus on the southern hydrological network formed by channels and ponds, and investigate for possible local introductions that have not yet been detected or reported.

### 4.3. Are Hydroacoustics and Environmental DNA Methods Equivalent, Complementary, or Synergists?

The patterns of *S. glanis* abundance in the Guadalquivir River Basin were assessed using hydroacoustics and catfish eDNA qPCR/ddPCR with a dual-mapping approach. Both techniques (hydroacoustics and eDNA molecular analyses) successfully detected catfish in the upper and lower reaches of the Guadalquivir River Basin. Moreover, both showed a significant decrease in the abundance patterns of *S. glanis* as one moves towards lower latitudes along the main channel of the river, scientifically confirming the extent of catfish invasion into the lower Guadalquivir River, reaching almost the marsh area (17 km downstream), just 57 km from Doñana National Park. On the other hand, a direct comparison of catfish detection/abundances inferred from the two techniques showed non-significant results. Hydroacoustic data seem to be connected to molecular results, though not within the typically reported range of 3 km from the eDNA sampling points [84], but rather at greater distances (>6 km). The limited overlap in sampling locations and lack of temporal coincidence between the surveys likely hindered the ability to validate one technique against the other. Despite the absence of a direct correlation between both methods, they should be considered synergistic, as their combined use offers a broader perspective on the presence of catfish in the river basin. Furthermore, the complementarity of these techniques provides valuable new information about the species’ distribution.

The study area borders and overlapping regions where both techniques were applied are interesting and complement the information on species dispersal. (1) An exclusive catfish eDNA detection at La Señuela (Figure 1) raises a serious concern regarding the southern hydrological network formed by channels and ponds in the southernmost areas surrounding the main Guadalquivir river bed. (2) An exclusive hydroacoustic survey carried out from the Guadaíra River intersection with the Guadalquivir to the locality of Coria del Río pointed to scarce or even no presence of catfish (Figure 1 and Figure S1). (3) Both techniques suggested an increasing gradient of catfish abundance from Coria del Río to La Rinconada (Figure 1), and (4) upstream of the Alcalá del Río dam the *S. glanis* presence is certainly abundant, as shown by both methodologies, including the uppermost areas sampled exclusively for eDNA, where relative abundances are similar to those observed at the invasion origin in the Iznájar Reservoir (Figure 1). Both techniques have proven useful and now serve as viable alternatives for future monitoring. The eDNA sampling method is easier, cheaper, and can detect catfish juveniles. However, while the eDNA detection methodology and assays are ready, the validation of fish abundance estimates is still a pending and challenging task that should be addressed promptly. Wide and navigable rivers or reservoirs would need more eDNA replications to foster accuracy. However, hydroacoustics, already demonstrated as a valid tool, can be a very practical solution in such areas.

### 4.4. Implications of the S. glanis Invasion Front in the Lower Guadalquivir

The results from hydroacoustic and eDNA surveys confirm the spread of invasive catfish in the lower Guadalquivir River, reaching almost the Doñana National Park, which is home to over 280 species of high ecological and economic value [11,85]. The lower Guadalquivir River is critical for the breeding and growth of commercial fish species [11,85], and the river course also serves as a migratory route for several species [11]. The Alcalá del Río dam represents a barrier that blocks migratory fish, which aggregate below the dam making them easy prey for catfish [86,87,88]. The introduction of catfish, a non-native apex predator [7,86], poses a serious threat to Iberian native freshwater species, which are not adapted to heavy predation [89,90,91]. Additionally, if the catfish spreads to the Doñana wetlands, it would not only threaten fish species but also the large waterfowl community in the area, due to its ability to prey on birds [92,93].

While the natural dispersal of catfish is generally slow, higher water temperatures can accelerate this process [94,95]. In the Iberian Peninsula, the species has spread rapidly in rivers like the Ebro and Tajo [35,96], due to several factors: its trophic plasticity, which allows it to exploit diverse habitats and resources, including surface areas; its high growth rate, enhanced by warm temperatures; and its physiological resilience [7,31,86,97,98]. These adaptations enable the species to thrive in degraded, polluted, and low-visibility environments, such as the highly turbid waters of the Guadalquivir River [99,100] where it holds a competitive advantage over other predators.

The stretch of the Guadalquivir River from the Alcalá del Río dam to its mouth exhibits distinctive hydrology, characterised by changes in salinity. This is influenced by a combination of factors such as tides, river flow, wind, precipitation, and drought periods. These conditions create a mobile ‘salt barrier’ that can extend as far as Coria del Río, the furthest point where catfish were detected in this study [99,101]. Laboratory experiments conducted by Krasteva et al. [102] suggest that this ‘salt barrier’ may represent a key obstacle limiting the expansion of the catfish. However, this barrier is mobile and shifts towards the river mouth during periods of rainfall and floods, or during irrigation seasons when water from various reservoirs in the basin is released to supply the rice fields, which are widely cultivated in the surrounding area. This could facilitate the entry of the species into areas within the Doñana National Park, leading to critical ecological consequences for the ichthyofauna and avifauna of this protected ecosystem.

The Guadalquivir estuary is biologically, ecologically, and economically unique, but its ecosystem could be disrupted as invasive catfish populations reach saturation [86]. Efficient tools are needed for monitoring the distribution of this species in real time. The non-invasive methods used in this study proved to be effective and align with previous research, showing that eDNA and hydroacoustics can reliably detect invasive species and estimate fish populations in aquatic environments.

## Data Availability

The original contributions presented in the study are included in the article/Supplementary Materials. Further inquiries can be directed to the corresponding author/s.

## Supplementary Materials

The following supporting information can be downloaded at: https://www.mdpi.com/article/10.3390/ani15020285/s1, Figure S1: Hydroacoustic survey; Figure S2: Environmental DNA sampling sites; Figure S3: Length frequency histogram; Figure S4: PCA analysis of environmental DNA quantification results and physico-chemical parameters; Figure S5: Environmental DNA quantification results; Figure S6: Correlation of environmental DNA quantification results; Table S1. Recorded average and maximum body size of fish species inhabiting the study area. Table S2: Physico-chemical data from locations sampled in this work for eDNA analyses Table S3: Results of the linear regression analyses for environmental parameters and molecular methods in rivers and reservoirs. SI.1: Detailed protocols for primers, qPCR and ddPCR designs and optimizations [60,103,104,105,106,107].
