# Supplementary material for: Environmental DNA and Hydroacoustic Surveys for Monitoring the Spread of the Invasive European Catfish (Silurus glanis Linnaeus, 1758) in the Guadalquivir River Basin, Spain
Source: Animals (Basel). 2025 Jan 20;15(2):285. doi: 10.3390/ani15020285 (PMC11761632; doi:10.3390/ani15020285)
Supplement: Supplementary file 1 [file animals-15-00285-s001.zip › 20122024-SuppMaterials.pdf]

SUPPLEMENTARY MATERIALS

Ruth Coya [1], Amadora Rodríguez-Ruiz [2], Álvaro Fueyo [1,3], Carlos Orduna [4], Laura Miralles [1], Ilaria de Meo [4], Trinidad Pérez [1], Juan Ramón Cid [4], Carlos Fernández-Delgado [5], Lourdes Encina [2,*], Yaisel J. Borrell [1,*] and Carlos Granado-Lorencio [2]

1 Department of Functional Biology, Genetics, University of Oviedo, 33006 Oviedo, Spain; coyaruth@uniovi.es (R.C.); afueyo@taxusmedioambiente.com (Á.F.); miralleslaura@uniovi.es (L.M.); pereztrinidad@uniovi.es (T.P.)
2 Department of Plant Biology and Ecology, University of Sevilla, 41012 Sevilla, Spain; dora@us.es (A.R.-R.); granado@us.es (C.G.-L.)
3 Environment and Sustainability Area, Taxus Medio Ambiente, 33006 Oviedo, Spain
4 EcoFishUS Research S.L.L., Sevilla, 41009, Spain; carlos@ecofishus.com (C.O.); ilaria@ecofishus.com (I.d.M.); juanramon@ecofishus.com (J.R.C.)
5 Department of Zoology, University of Cordoba, 14071 Cordoba, Spain; ba1fedec@uco.es
* Correspondence: lencina@us.es (L.E.); borrellyaisel@uniovi.es (Y.J.B.)

Figures

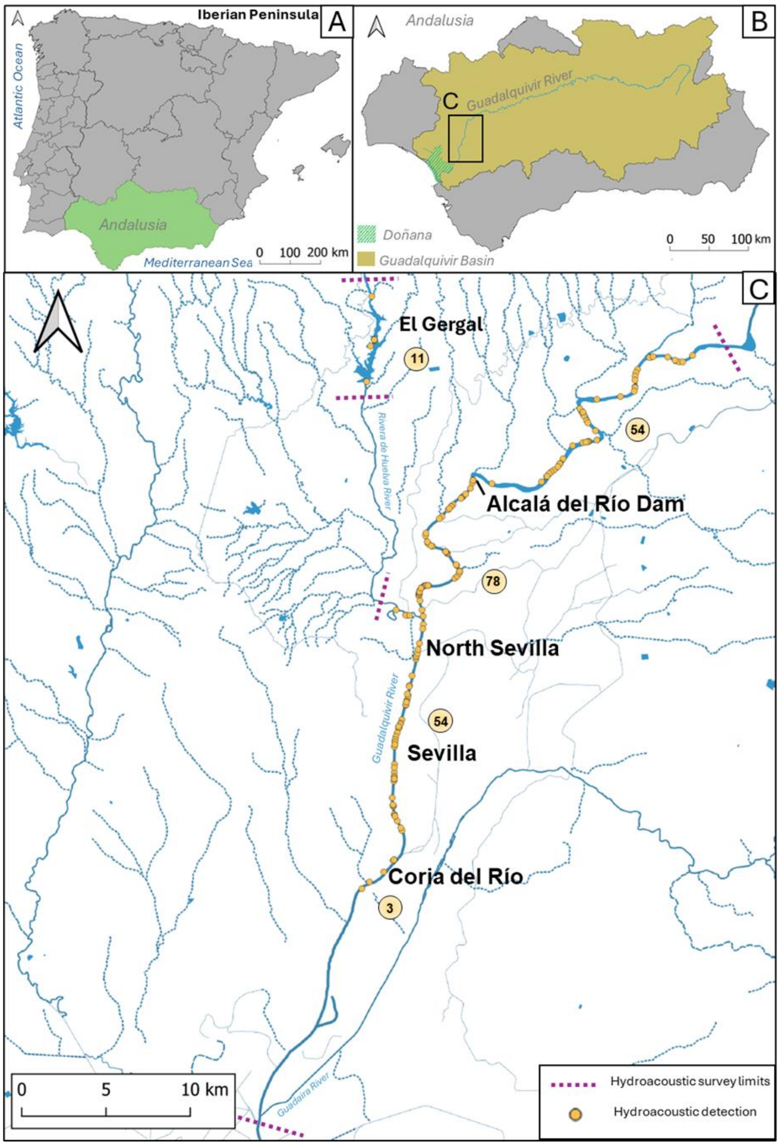

Figure S1. Hydroacoustic survey. Detection of *Silurus glanis* results of the Hydroacoustic surveys in the Guadalquivir Basin. (A) Iberian Peninsula showing the Andalusian region. (B) The Andalusian region showing the Guadalquivir Basin and including the Doñana National Park location. (C) The detailed Guadalquivir Basin in province of Sevilla showing the section sampled for hydroacoustics with a purple dotted line. Each individual is displayed as a yellow dot (some of them overlap) and the number of total individuals per section described is displayed inside a yellow dot. Alcalá del Río Dam is indicated with a black line.

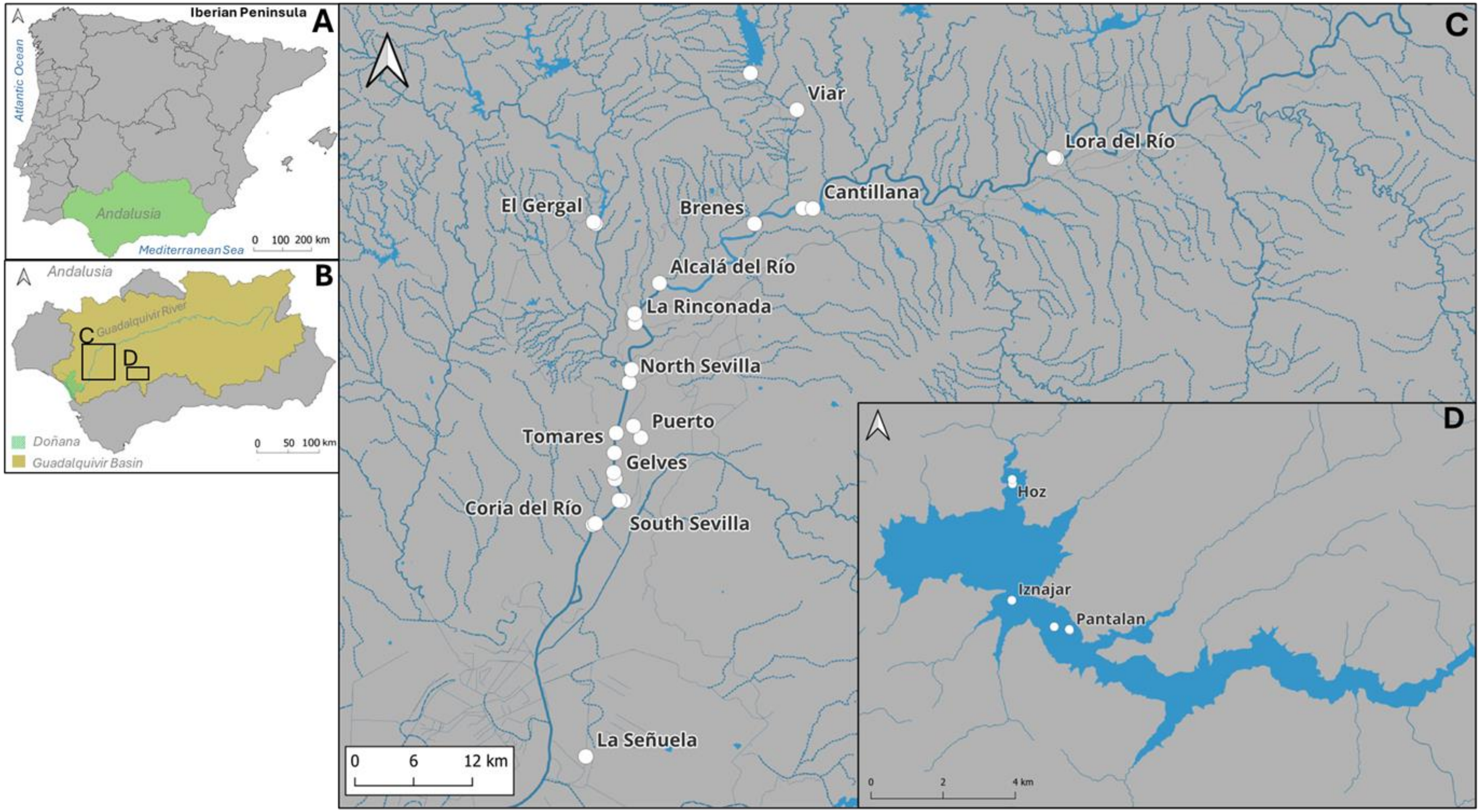

Figure S2. Environmental DNA sampling sites. (A) Iberian Peninsula showing the Andalusian region. (B) The Andalusian region showing the Guadalquivir Basin and including the Doñana National Park location. (C) The detailed Guadalquivir Basin in province of Sevilla. (D) Detailed

map of the Iznájar Reservoir in the Guadalquivir River Basin, province of Córdoba. White dots indicate the location of environmental DNA sampling. Two water samples were taken per point.

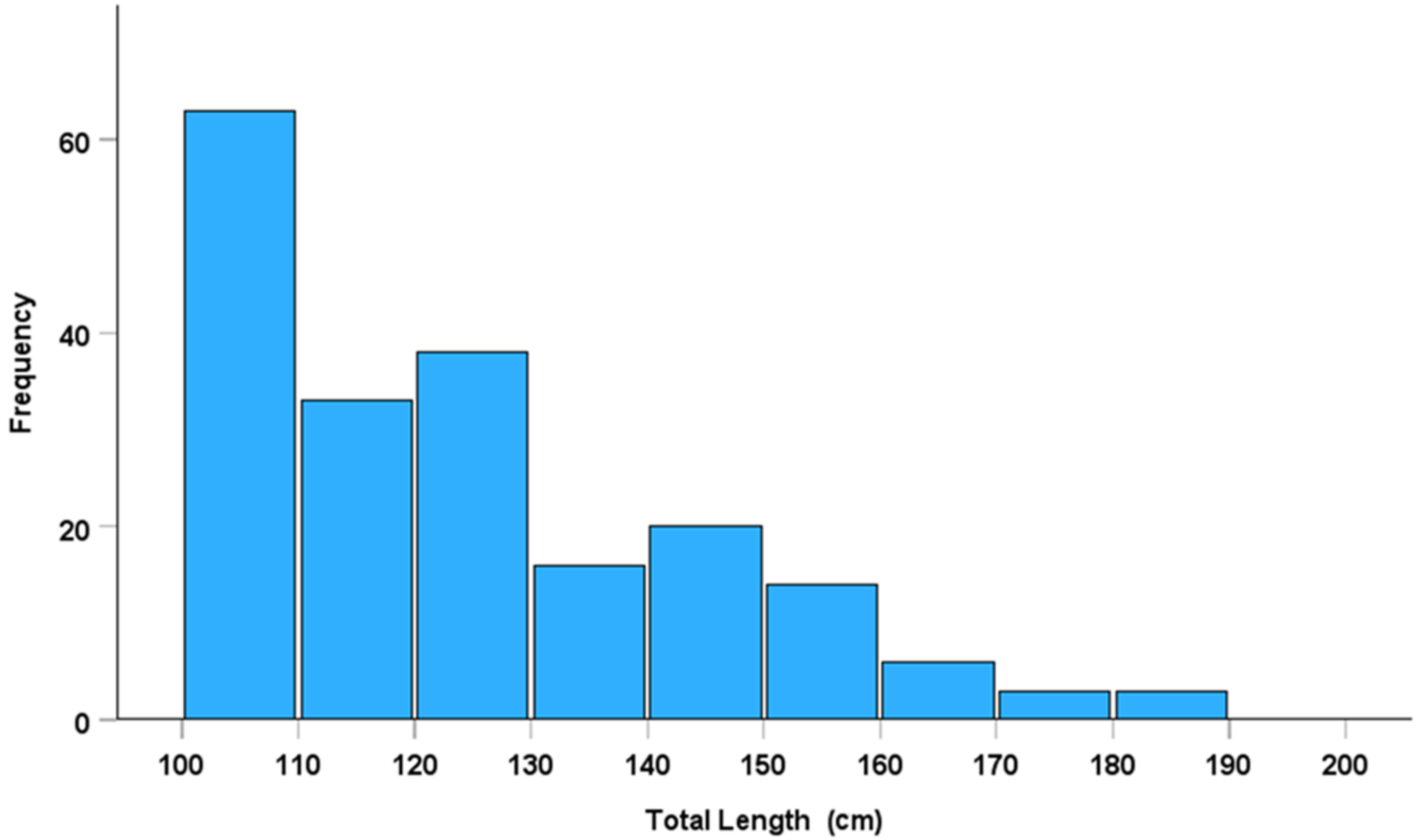

Figure S3. Length frequency histogram. Total Length (cm) of *Silurus glanis* specimens detected in the surveyed area by hydroacoustic.

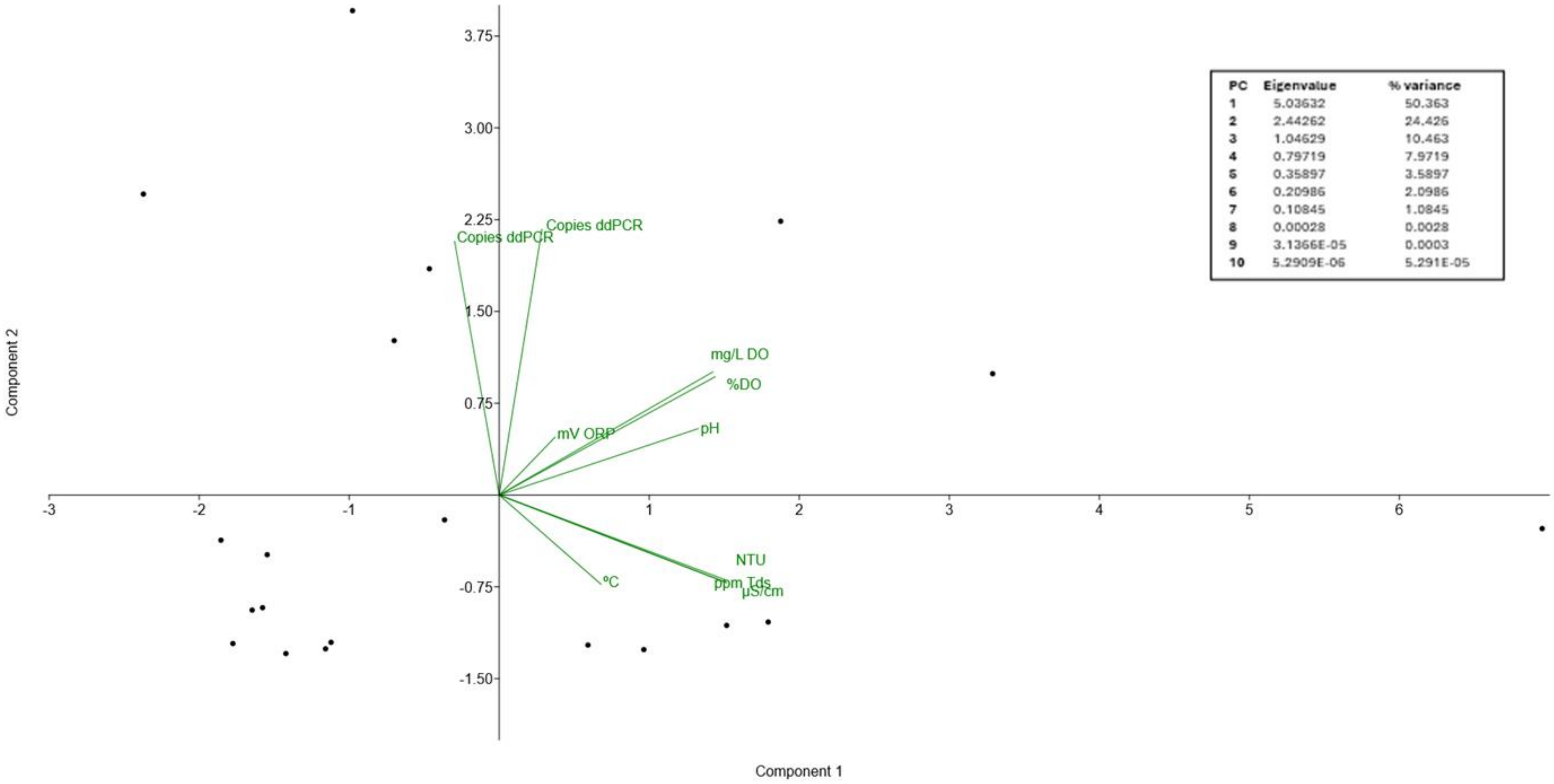

PC	Eigenvalue	% variance
1	5.03632	50.363
2	2.44262	24.426
3	1.04629	10.463
4	0.79719	7.9719
5	0.35897	3.5897
6	0.20986	2.0986
7	0.10845	1.0845
8	0.00028	0.0028
9	3.1366E-05	0.0003
10	5.2909E-06	5.291E-05

Figure S4. PCA analysis of Environmental DNA quantification results and physico-chemical parameters. Principal Component Analysis (PCA) biplot based on the correlation matrix and calculated from the values of catfish DNA concentrations from qPCR/ddPCR in 2 water liters and physicochemical parameters.

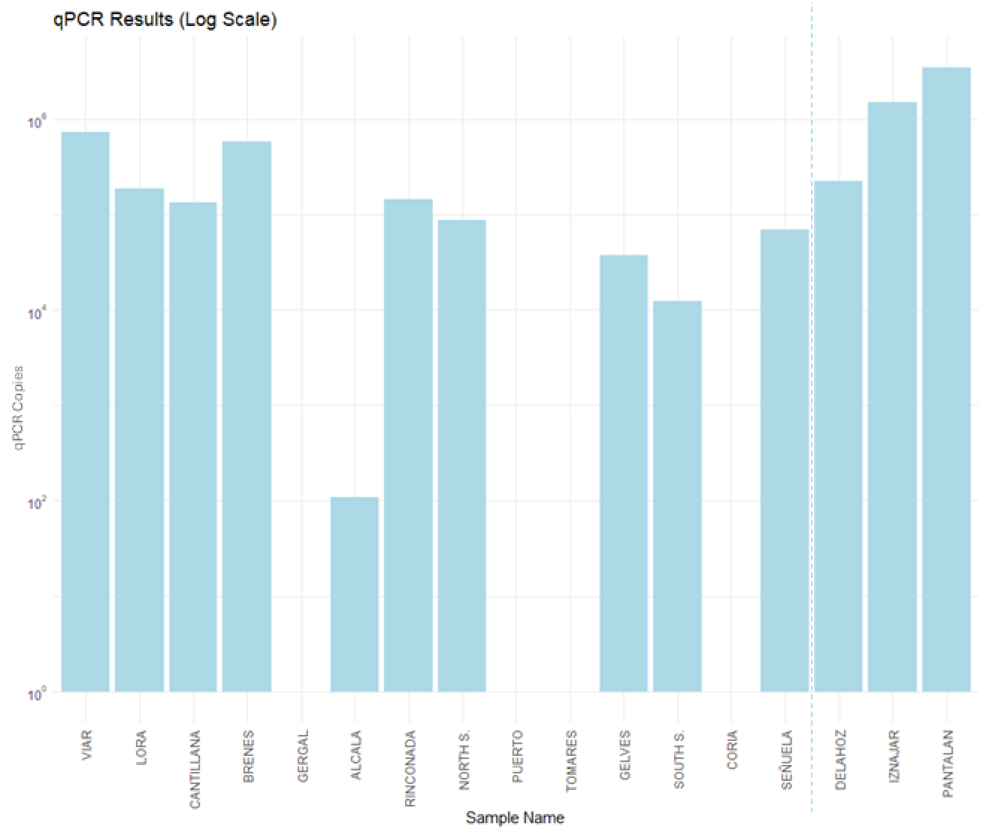

qPCR Results (Log Scale)
qPCR Copies
10^6
10^4
10^2
10^0
VIAR
LORA
CANTILLANA
BRENES
GERGAL
ALCALA
RINCONADA
NORTH S.
PUERTO
TOMARES
GELVES
SOUTH S.
CORIA
SEÑUELA
DELAHOZ
IZNAJAR
PANTALAN
Sample Name

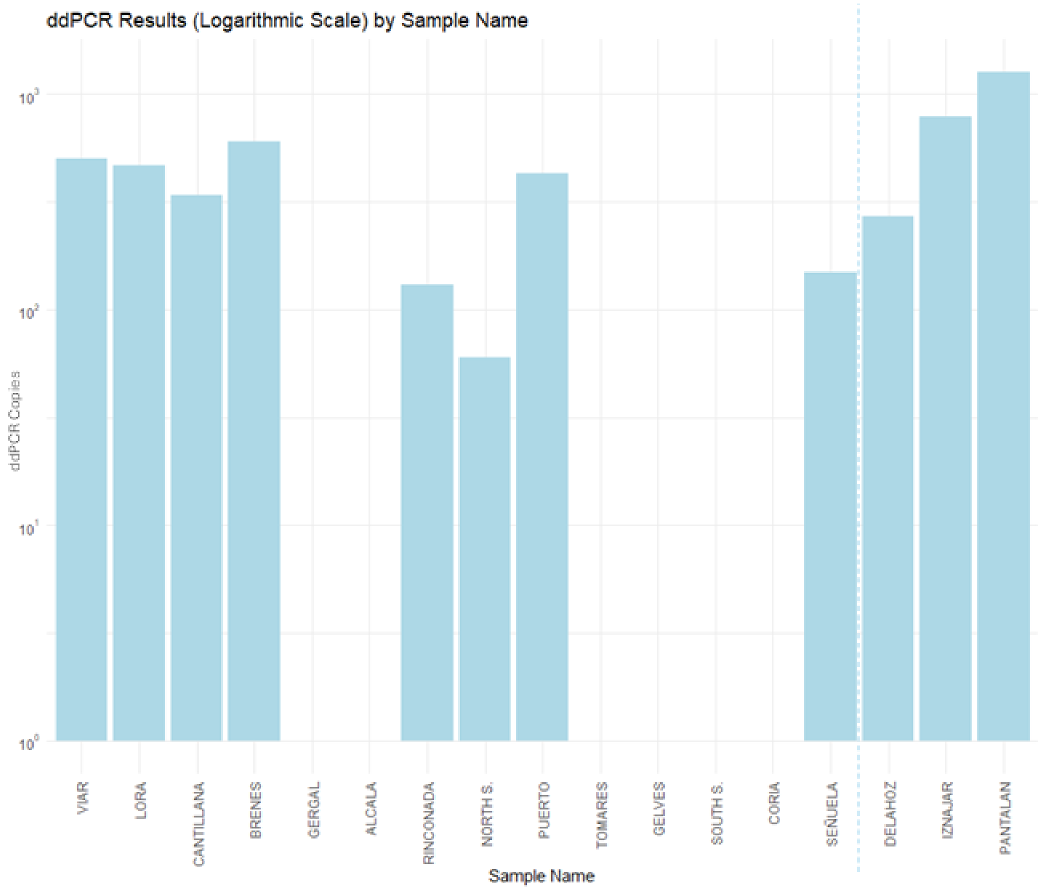

Figure S5. Environmental DNA quantification results. *Silurus glanis* copy numbers by site in logarithmic scale in environmental DNA obtained for qPCR and ddPCR assays in 2 water liters. Samples are ordered from north to south latitude. Positive controls from the Iznajar reservoir area are separated on the right side.

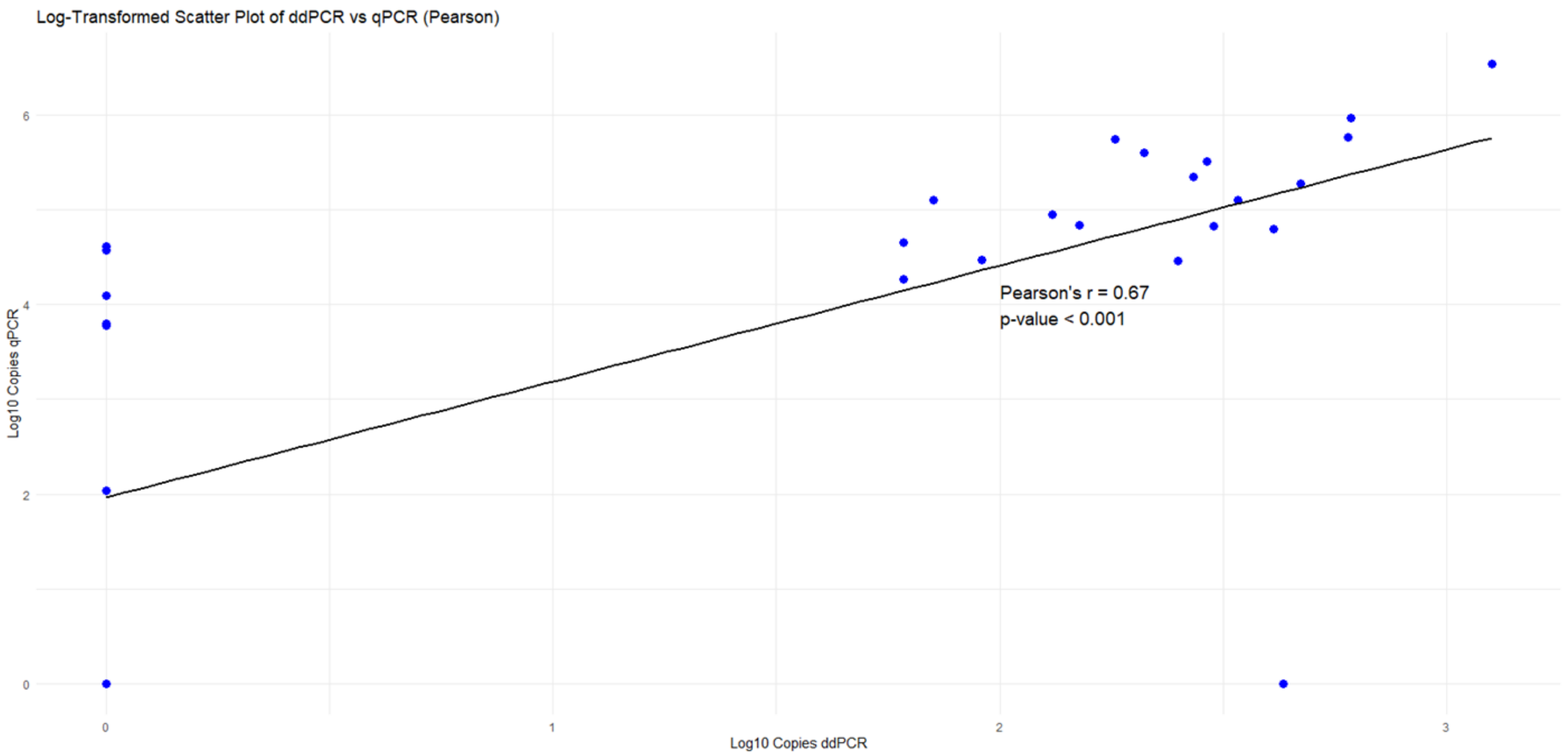

Figure S6. Correlation of Environmental DNA quantification results. *Silurus glanis* copy numbers in environmental DNA obtained for qPCR and ddPCR assays in 2 water liters scatterplot using Pearson correlation coefficient with log-transformed data

111. Kassambara, A. (2020). ggpubr:"ggplot2" based publication ready plots (R package version 4.3.3). *ggpubr:'ggplot2'based publication ready plots (R Package Version 4.3.3)*

112. Wickham, H., & Wickham, H. (2016). *Data analysis* (pp. 189-201). Springer International Publishing.

Tables

Table S1. Recorded average and maximum body size of fish species inhabiting the study area. FL: Furcal length. TL: Total length.

Species	Mean length (cm)	Maximum length (cm)
Luciobarbus sclateri	FL: 11.0 [51]	FL: 62.0 [51]; TL: 40 [6]
Cyprinus carpio	FL: 12.3 [51]	FL: 45.0 [51]
Carassius gibelio	FL: 9.6 [51]	FL: 28.4 [51]
Chelon ramada	FL: 16.7 [51]	FL: 44.0 [51]; TL: 48.7 [50]
Mugil cephalus	FL: 12.2 [51]	FL: 35.0 [51]
Alosa alosa	TL: 66.9 [52]	TL: 83.0 [6]; TL: 74 [52]
Alosa fallax	TL: 45.7 [52]	TL: 55.0 [6]; TL: 56 [52]
Micropterus salmoides	FL: 12.4 FL [51]	FL: 27.8 [51]
Anguilla anguilla	FL: 36.2 FL [51]	FL: 68.0 [51]

6. Doadrio, I. Atlas y Libro Rojo de Los Peces Continentales de España.; Dirección General de Conservación de la Naturaleza. Museo Nacional de Ciencias Naturales (CSIC), Ed.; Madrid, España., 2001; ISBN 84-8014-313-4.
50. Oliveira, J.M.; Ferreira, T. Abundance, Size Composition and Growth of a Thin-Lipped Grey Mullet, Liza Ramada (Pisces: Mugilidae) Population in an Iberian River. Folia Zool Brno 1997, 46(4), 375–384
51. Fernández-Delgado, C. La Zona de Cría y Engorde Del Bajo Guadalquivir. In El río Guadalquivir, del mar a la marisma, Sanlúcar de Barrameda; Javier Rubiales Torrejón, Ed.; Junta de Andalucía, Consejería de Obras Públicas y Transportes, 2011; Vol. 2, pp. 89–95.
52. Nachón, D.J.; Mota, M.; Antunes, C.; Servia, M.J.; Cobo, F. Marine and Continental Distribution and Dynamic of the Early Spawning Migration of Twaite Shad (Alosa Fallax (Lacépède, 1803)) and Allis Shad (Alosa Alosa (Linnaeus, 1758)) in the North-West of the Iberian Peninsula. Mar Freshw Res 2016, 67, 1229, doi:10.1071/MF14243.

Table S2. Physico-chemical data from locations sampled in this work for eDNA analyses.

NAME	Latitude	Longitude	Alkalinity pH	Redox potential mV	Dissolved oxygen percentage %	Dissolved oxygen concentration mg/L	Conductivity µS/cm	Total Dissolved Solids ppm	Turbidity NTU	Temperature ºC
BRENES	37,575911	-5,868484	8.18	29.5	76.1	5.90	822	411	0.40	28.41
CANTILLANA-R1	37,591843	-5,813950	7.85	29.5	76.7	5.94	1211	605	0.60	28.46
CANTILLANA-R2	37,591843	-5,802226	8.00	26.6	87.7	6.75	1260	630	0.62	28,88
VIA-R1	37,714754	-5,879191	7.99	27.6	87.9	6.76	1260	630	0.62	28.91
VIAR-R2	37,682358	-5,824086	7.66	48.3	51.6	4.10	348	174	0.16	27.02
LORA DEL RÍO	37,644520	-5,522099	8.71	43.6	128.7	9.47	1473	736	0.73	31.18
LA SEÑUELA	37,080998	-6,044376	9.04	32.8	1900	13.97	5356	2678	2.86	30.64
PUERTO	37,376075	-5,992755	9.16	25.3	121.1	9.28	3229	1615	1.68	28.57
GELVES-R1R2	37,337257	-6,020870	7.63	27.5	26.9	2.06	1794	897	0.90	29.10
TOMARES-R1	37,361304	-6,022564	7.84	25.1	32.3	2.47	1555	777	0.78	29.22
TOMARES-R2	37,379637	-6,021420	7.74	30.1	20.0	1.53	1480	740	0.74	29.3
NORTHSEVILLA-R1	37,426600	-6,008430	7.78	31.0	16.7	1.23	1173	586	0.58	29.83
NORTHSEVILLA-R2	37,438626	-6,005918	7.70	29.4	14.8	1.12	1175	588	0.58	29.85
LA RINCONADA-R1	37,481536	-6,003248	7.72	36.2	20.8	1.57	1122	561	0.55	29.93
LA RINCONADA-R2	37,490135	-6,004488	7.80	14.2	38.3	2.92	889	434	0.42	29.47
SOUTHSEVILLA-R1	37,317527	-6,010229	8.04	31.5	54.1	4.13	2656	1328	1.36	29.05
SOUTHSEVILLA-R2	37,317743	-6,015439	8.06	33.3	62.9	4.77	2747	1373	1.41	29.44
CORIA DEL RÍO-R1	37,295845	-6,041935	8.34	41.3	72.8	5.53	3149	1574	1.63	29.04
CORIA DEL RÍO-R2	37,294605	-6,044637	8.11	40.7	74.3	5.64	3018	1509	1.56	29.15
ALCALÁ DEL RÍO	37,518833	-5,976858	8.63	12.4	37.3	2.89	763	381	0.37	28.32
EL GERGAL	37,573170	-6,055339	8.99	48.8	111.4	8.77	294	147	0.14	27.65
IZNAJAR-R1	37,262466	-4,331253	7.88	40.3	70.8	5.83	1405	702	0.70	22.77
IZNAJAR-R2	37,268924	-4,344617	8.40	50.0	115.3	8.97	1525	733	0.82	22.95
PANTALAN	37,261708	-4,326423	8.45	40.8	105.4	8.34	2035	1056	0.85	28.25
HOZ	37,299141	-4,345061	8.32	33.9	101.9	7.99	2492	1265	0.87	28.95

Table S3. Results of the linear regression analyses for environmental parameters and molecular methods in the Guadalquivir Basin rivers and reservoirs.

	**RIVERS**				
**ddPCR**	**Predictor**	**Estimate**	**Std. Error**	**t-value**	**p-value**
	(Intercept)	16.456	0.9060	1.816	0.087002
	mg/L DO	0.7755	0.1920	4.039	**0.000853** ***
	NTU	-30.163	0.9842	-3.065	**0.007015** **
	**Adjusted R^2 = 0.43, F-statistic = 8.322, p = 0.003**				
	**RESERVOIRS**				
**qPCR**	**Predictor**	**Estimate**	**Std. Error**	**t-value**	**p-value**
	(Intercept)	235.248	72.589	3.241	**0.0119***
	NTU	84.686	27.994	3.025	**0.0164***
	**Adjusted R^2 = 0.47, F-statistic = 5.4, p = 0.032**				
