# Supplementary material for: Environmental DNA and Hydroacoustic Surveys for Monitoring the Spread of the Invasive European Catfish (Silurus glanis Linnaeus, 1758) in the Guadalquivir River Basin, Spain
Source: Animals (Basel). 2025 Jan 20;15(2):285. doi: 10.3390/ani15020285 (PMC11761632; doi:10.3390/ani15020285)
Supplement: Supplementary file 1 [file animals-15-00285-s001.zip › 22122024-Final-SupportingInformation_SI1.pdf]

__SUPPLEMENTARY INFORMATION (SI.1).__

DETAILED PROTOCOLS FOR PRIMERS, qPCR AND ddPCR DESIGNS AND OPTIMIZATIONS.

I. Primers design and validation

Complete mitochondrial sequences from all the species of the genus *Silurus* and other co-occurring species were retrieved from the National Center for Biotechnology Information (NCBI) nucleotide database. The Primer3 software implemented in Geneious Prime® 2023.1.1 (Kearse *et al*., 2012) was used for primers and probe design. Primer3 parameters were established to optimize the design of eDNA and qPCR primers. As all species of the genus *Silurus* that might be present in this environment are considered invasive, *Silurus* genus sequences were used as sequences to amplify. TaqMan® primers and probes were designed to target any species of the genus *Silurus.* As a result, two specific primers and a probe (FAM-labeled) were developed for a fragment of the 12S rRNA mitochondrial gene of the genus *Silurus*: Sil1-F (5'-TTTTCCCCGCCTATATACCGCC-3') Sil1-R (5'-CTTCGGGCACTTACTTTCAAGG-3') and Sil1-probe (**FAM-AACGTCAGGTCGAGGTGTAGCGTACG-MGB). Cross amplifications of untargeted species were verified using Primer Blast (Ye *et al*., 2012).

This primer pair and probe were then tested in vitro by PCR on European catfish *S. glanis* and on *S. aristotelis* (Garman, 1980) tissue samples used in Parrondo *et al.* (2018). DNA was extracted using the QIAGEN QIAamp DNA Mini Kit (Tissue Protocol) (Qiagen, Hilden, Germany) according to the manufacturer's instructions and stored at -20ºC. All the individuals were barcoded using the cytochrome oxidase subunit I (COI) gene (Ward *et al*., 2005). Genetic identifications were performed using the BOLD system identification engine (http://www.boldsystems.org/index.php/IDS_OpenIdEngine), and species identifications were only accepted greater than 98% identity (Parrondo *et al*., 2018).

The designed primers were subjected to a thorough screening process to confirm their specificity, and to test the potential for cross-amplification with several non-target species prevalent in the region, both native and invasive (*Pseudorasbora parva* (Temminck & Schlegel, 1846); *Scardinius erythrophthalmus* (Linnaeus, 1758); *Leuciscus idus* (Linnaeus, 1758); *Alburnus alburnus* (Linnaeus, 1758); *Phoxinus Phoxinus* (Linnaeus, 1758); and *Lepomis gibbosus* (Linnaeus, 1758)). In addition, three environmental samples (5L each) were collected from the Nora River (Asturias, Spain), where catfish is not present. The *S. glanis* tissue (0.5g) was added overnight in two of the water samples, while the other, used as a negative control, did not contain tissue. The inclusion of tissue prior to filtration and extraction of eDNA is intended to replicate the occurrence of the target species in water samples.

A preliminary primer screening was performed in a conventional PCR using GoTaq® G2 Flexi DNA Polymerase Kit (Promega Corporation, Madison, WI, USA) on a VeritiPro™ Thermal Cycler (Applied Biosystems) in a final volume of 20 µl: 4 µl of 1X

Green GoTaq® Flexi Buffer, 2 µl dNTPs (EURx®, Poland) (0.25 mM each) 2 µl of $MgCl_2$ (25 mM), 0.5 µl of each primer (10 µM), 0.5 U of Go Taq® G2 Flexi Polymerase, 1 µl of DNA and completed with ultrapure ddH_2O, following these conditions: 95°C for 5 min, 35 cycles × [98°C for 30 s, 60°C for 30 s, 72°C for 30s] and a final extension for 7 min at 72°C. The PCR products were then visualized on a 2% electrophoresis agarose gel. Primer pairs that showed strong amplification of the target and no amplification of the non-target species were selected. To estimate the detection sensitivity, 10-fold serial dilutions were used, starting from 8 ng/µl down to 1:1,000,000 and the limits of detection were defined by PCR amplification.

II. Optimization and Quantification by qPCR

To estimate the detection sensitivity of the specific primer pair and probe a standard curve with 7 serial dilutions of catfish DNA was used in qPCR assay. This was done to assess the effectiveness of the assays under conditions similar to their prescribed use, and has been previously validated (Yun *et al.*, 2019; Benoit *et al.*, 2023). The curve was added to each plate in all assays performed. For the generated standard curves ($y = 3.16x + 15.18$, $y = 3.27x + 14.06$, $y = 3.1x + 15.61$, $y = 3.49x + 13.5$), the assays showed an amplification efficiency ranging between 93.5% and 110%. As expected, catfish DNA was successfully amplified and an efficiency considered acceptable was achieved (Taylor *et al.*, 2019), and all curves obtained an $R^2=0.99$.

All qPCR reactions were conducted in a dedicated pre-PCR laboratory where bench surfaces were sterilized with UV radiation. Each PCR contained 6 µl of template DNA, 1.2 µL of each primer (10 µM), 0.8 µL of probe (5 µM), 10 µl of TaqMan® Environmental Master Mix 2.0 (Thermo Fisher Scientific) and DNase/RNase-free water to a final volume of 20 µl. Each PCR plate contained a standard curve consisting of a 1:10 dilution series to generate a standard curve of known amounts of DNA. Three technical (PCR) replicates were performed for each biological sample, resulting in a total of 12 replicates per sampling point. In addition, 3 no template controls (NTCs) per plate were included, as well as filtration and extraction controls. The filtration, extraction and technical controls returned negative results. Samples were run on an Applied Biosystems 7900HT Fast Real-Time PCR System (Thermo Fisher Scientific) under the following thermal cycling conditions: 2 minutes at 50°C, 10 minutes at 95°C, 50 cycles of 15 seconds at 95°C, and 60 seconds at 60°C. Sequence Detection Software SDS 2.4 (Thermo Fisher Scientific) was used to analyze the results.

III. Optimization and Quantification by ddPCR

Samples were analyzed at the Genomics Service of the Scientific and Technical Services of the Autonomous University of Barcelona (SGiEB) using the ddPCR assay with primers and probes designed in this study. In this instance, filtration replicates per

sampling point were consolidated, yielding a total of 46 samples, each assay comprising 2 technical replicates.

Each ddPCR reaction mix (20 µL) comprised 10 µL of sample, 900 nM of each primer, and 250 nM of TaqMan probe, completed with Supermix for Probes (No dUTP) (1863023) (Bio-Rad, Hercules, CA, USA) at a concentration of 1×. This mixture was then combined with Bio-Rad's droplet generation oil and divided into 15,000–20,000 droplets using the QX-100 droplet generator (Bio-Rad). Individual sample droplets were separately to each well of a 96-well PCR reaction plate. PCR was performed in the sealed 96-well plate using the Bio-Rad T-100 thermocycler (Bio-Rad). The optimal primer annealing temperature for ddPCR was determined using a gradient PCR and 57°C was found to be the best annealing temperature, with the highest separation between positive and negative droplets. The final PCR conditions were 10 minutes at 95°C, 40 cycles of denaturation for 30 seconds at 94°C, and extension for 90 seconds at 57°C with a temperature ramp of 2ºC/second, followed by 10 minutes at 98°C, and a hold at 4°C until plate reading. After PCR amplification, the plate was transferred to the Bio-Rad QX-200 droplet reader (Bio-Rad).

Bio-Rad's QuantaSoft software, version 1.7.4.0917, was used to quantify copies of the target DNA. The threshold for a positive signal was determined according to the QuantaSoft instructions. Each droplet with a signal above the fluorescence threshold was counted as a positive event. Technical ddPCR negatives showed negative results for DNA copies.

References

35. Parrondo, M.; Clusa, L.; Mauvisseau, Q.; Borrell, Y.J. Citizen Warnings and Post Checkout Molecular Confirmations Using eDNA as a Combined Strategy for Updating Invasive Species Distributions. *J. Nat. Conserv.* **2018**, *43*, 95–103, doi:10.1016/j.jnc.2018.02.006.

59. Kearse, M.; Moir, R.; Wilson, A.; Stones-Havas, S.; Cheung, M.; Sturrock, S.; Drummond, A. Geneious Basic: An Integrated and Extendable Desktop Software Platform for the Organization and Analysis of Sequence Data. *Bioinformatics* **2012**, 28, 1647–1649, doi:10.1093/bioinformatics/bts199.

103. Benoit, N.P.; Robinson, K.M.; Kellogg, C.T.; Lemay, M.A.; Hunt, B.P. Using qPCR of Environmental DNA (eDNA) to Estimate the Biomass of Juvenile Pacific Salmon (*Oncorhynchus* spp.). *Environ. DNA* **2023**, 5, 683–696, doi:10.1002/edn3.422.

105. Taylor, S.C.; Nadeau, K.; Abbasi, M.; Lachance, C.; Nguyen, M.; Fenrich, J. The Ultimate qPCR Experiment: Producing Publication Quality, Reproducible Data the First Time. *Trends Biotechnol*. **2019**, 37, 761–774, doi:10.1016/j.tibtech.2018.12.002.

106. Ward, R.D.; Zemlak, T.S.; Innes, B.H.; Last, P.R.; Hebert, P.D.N. DNA Barcoding Australia's Fish Species. *Philos. Trans. R. Soc. B* **2005**, 360, 1847–1857, doi:10.1098/rstb.2005.1716.

107. Ye, J.; Coulouris, G.; Zaretskaya, I.; Cutcutache, I.; Rozen, S.; Madden, T.L. Primer-BLAST: A Tool to Design Target-Specific Primers for Polymerase Chain Reaction. *BMC Bioinform.* **2012**, 13, 134, doi:10.1186/1471-2105-13-134.

108. Yun, J.J.; Heisler, L.E.; Hwang, I.I.; Wilkins, O.; Lau, S.K.; Hyrcza, M.; Der, S.D. Genomic DNA Functions as a Universal External Standard in Quantitative Real-Time PCR. *Nucleic Acids Res*. **2006**, 34, e85, doi:10.1093/nar/gkl400.
